# Modeling a flexible staff scheduling problem in the Era of Covid-19

**DOI:** 10.1007/s11590-021-01776-3

**Published:** 2021-07-12

**Authors:** Francesca Guerriero, Rosita Guido

**Affiliations:** DIMEG, University of Calabria, Rende, CS Italy

**Keywords:** Staff scheduling, Resource management, Integer programming formulation, Covid-19

## Abstract

In this paper, we propose optimization models to address flexible staff scheduling problems and some main issues arising from efficient workforce management during the Covid-19 pandemic. The adoption of precautionary measures to prevent the pandemic from spreading has raised the need to rethink quickly and effectively the way in which the workforce is scheduled, to ensure that all the activities are conducted in a safe and responsible manner. The emphasis is on novel optimization models that take into account demand requirements, employees’ personal and family responsibilities, and anti-Covid-19 measures at the same time. It is precisely considering the anti-Covid-19 measures that the models allow to define the working mode to be assigned to the employees: working remotely or on-site. The last optimization model, which can be viewed as the most general and the most flexible formulation, has been developed to capture the specificity of a real case study of an Italian University. In order to improve employees’ satisfaction and ensure the best work/life balance possible, an alternative partition of a workday into shifts to the usual two shifts, morning and afternoon, is proposed. The model has been tested on real data provided by the Department of Mechanical, Energy and Management Engineering, University of Calabria, Italy. The computational experiments show good performance and underline the potentiality of the model to handle worker safety requirements and practicalities and to ensure work activities continuity. In addition, the non-cyclic workforce policy, based on the proposed workday organization, is preferred by employees, since it allows them to better meet their needs.

## Introduction

The Corona Virus Disease 2019 is a new coronavirus first identified in Wuhan City, China, in January 2020. It has spread quickly all over the world causing a large number of deaths (the official data reported by the World Health Organization are: 235 countries, areas or territories with cases; 40.114.293 confirmed positive cases; 1 114 692 deaths. https://www.who.int/emergencies/diseases/novel-coronavirus-2019, Last access 20 October 2020). Following the Covid-19 pandemic, there have been several measures to fight the spread of the disease. Several nations adopted restrictions, such as suspension of non-essential activities and a substantial proportion of workers is continued to work from home. Currently, national administrations are authorising a gradual resumption of work activities. Obviously, all people must play a role in order to protect families, workers, and society at large. Appropriate preventive measures help to achieve a safe and healthy return to work. Covid-19 is known to be highly contagious [9, 15].

The measures, aimed at minimizing the exposure to Covid-19 at work, adopted in Italy can be found at the website of the Italian Government http://www.governo.it/it/coronavirus-misure-del-governo. For instance, regarding the education sector, the majority of the Italian Universities have closed to all staff and students but they continued to work from home using alternatives to on-campus working and teaching. Even though the social distancing represents the most critical issue for employers throughout the phases of re-opening, other rapidly changing staffing needs specific to Covid-19 have to be taken into account. For example, an employee can require to stay home to care for a sick family member or to respond to school/childcare closures. Firms and Universities should be then flexible even with their staff and should consider that every department has specific scheduling needs for its workforce. Employees’ personal and family responsibilities can affect their availability and scheduling needs. Better scheduling leads to higher employee satisfaction.

Basic workforce scheduling problems consist in assigning employees to shifts or days-off for a given period of time. The paper-tutorial [4] describes the main constraints to be handled in staff scheduling problems. There exist two main variants of this problem: rotating (or cyclic) workforce schedules and non-cyclic workforce schedules. In cyclic workforce schedules, there are three types of problems [2]: days off scheduling, shift scheduling, and tour scheduling. The days off scheduling problems aim to assign the workers work and non-work days over the planning horizon; the shift scheduling problems deal with determining the actual shifts during the planning horizon; the tour scheduling problems combine the two above problems. The tour scheduling problem in a multi-activity context is a very challenging problem recently addressed in [18]. The authors solve an optimization model, that minimizes under and over coverage, by taking into account workload demand and legal constraints. [20] focuses on a monthly tour scheduling problem with mixed skills, considering the weekend off requirements in contrast to the weekly planning horizon, that is typical in most of the published contributions on this topic. A general integer programming model and a binary integer programming formulation were developed. Numerical results showed that the second formulation is more effective than the first one. As reported in [16], employee scheduling problems have attracted significant attention in both research and practice [3, 7, 11] and they have been extensively studied in the literature. We cite, for instances, the survey papers [1, 6, 8, 10, 14, 17, 22], that review staff scheduling problems in specific application areas, analyzing models and algorithms. A comprehensive annotated bibliography of some 700 papers in this area, according to the type of problem addressed, the application areas covered and the used computational methods for rostering and personnel scheduling, is due to [13]. [12] formulates an optimization model, based on a generalised set-covering formulation, to schedule multi-skilled employees. Under-staffing and over-staffing are allowed. The goal is to minimise the total number of understaffed shifts. The authors show that instances with single skill are solved very quickly, by all the proposed methods, whereas the computational time increases when the number of skills per employee rises. [21] presents a goal programming model that implicitly represents scheduling flexibility and incorporates information about employees working patterns preferences. The model produces optimal solutions in very short computing times.

Despite the numerous papers in the literature, the level of employee’s satisfaction, mainly part-time, is not always considered in workforce scheduling models. Papers addressing these specific requirements are often devoted to nurse scheduling problems. Moreover, the current pandemic era requires changes in staff management and scheduling and calls for more flexible organisations. To the best of our knowledge, no papers addressed the staff scheduling problems in the Covid-19 Era, to date, with the only exception of the recent paper [23]. The authors propose a mixed integer linear programming formulation (MILP) for a large Italian pharmaceutical distribution warehouse. Employees are divided into mutually exclusive groups to prevent virus transmission. A schedule has to meet the contractual working time of employees. The goal is to minimize the total deviation between the amount of weekly contractual hours for each worker and the actual working hours. The schedule that the authors found in the case study outperforms the solution generated by the company. Computational tests performed on random instances of larger size showed high- quality solutions even using an open-source MILP solver within an acceptable CPU time.

The real-life personnel scheduling problem here addressed embraces basically the following challenges: designing the most suitable schedule for employees (shifts, duties, employees availability and preferences) in the Covid-19 Era and, at the same time, meeting the demand requirements.

We focus on non-continuous workforce schedules and there are no night shifts for staff. This is typical, for instance, in case of the office jobs. We formulate six optimization models to address tour scheduling problems in the Covid-19 Era. The proposed mathematical formulations are characterized by an increasing level of flexibility and have been defined in such a way as to capture some of the relevant features of the specific scenario under study. We consider multiple-shift scheduling (i.e., each day is divided into either only two or several shifts) and a regular work schedule based on five-day workweeks. The specific cases of overlapping/non-overlapping and compatible/incompatible shifts [17, 22] are also taken into consideration.

The proposed optimization models can be classified as flexible workforce scheduling problems [8], because they consider different working times and both employee availability and preferences. The aim is to maximize employee’s satisfaction and meet the demand requirements for each day.

The first model is a bi-objective optimization formulation, aimed at maximizing the demand requirements satisfaction, by considering the availability of employees to work. The objective functions of the problem are conflicting [5], and the problem can be infeasible in some cases, due to the presence of hard constraints related to the employees’ availability. The main goal is to support the staff manager in deciding when an employee works, i.e., which day and which shift, and where, that is, in office or at home.

The other models are single-objective formulations, where employee’s preferences are taken into consideration instead of the employees’ availability, by introducing soft constraints. The main aim is to find schedules, ensuring the best trade-off between employees’ needs and companies’ requirements.

The last proposed mathematical model, characterized by the highest degree of flexibility, has been defined in such a way to handle in an appropriate way the requirements of an Italian University, considered as a case study. To this aim, some features, not included in the other formulations, such as overlapping shifts, incompatible shifts, and flexibility for parents, have been introduced. Computational experiments on real data have been carried out to evaluate empirically the performance of the most flexible versions of the proposed mathematical models. The rest of the paper is organized as follows. Section 2 presents the main features of the scenario under study. Section 3 gives a detailed description of the proposed optimization models, and shows how more flexible models can be derived, starting from the basic formulations. Section 4 introduces the real case study and discusses on the schedules found by solving the proposed optimization models. Computational results obtained on larger-size instances are also presented. Finally, Sect. 5 concludes the paper.

## Problem description

In this section, the problem description and the made assumptions are presented in order to formulate the required integer programming models reported in Sect. 3.

The Covid-19 pandemic caused shifts in workforce. Several professional services sectors were forced to work remotely from home. For instance, in the majority of Italian Universities staff works remotely, as the containment measures applied by national government and local authorities required, to reduce the spread of the Covid-19 virus. The university’s priorities are to design procedures to:

- Assure the safety and health of students, faculty, and staff;
- Maintain, as it is possible, student services, academic courses, and administrative work;
- Define flexible models to help ensure the well-being of the entire academic community.

Universities, as well as companies, are going to adopt strategies like organizing smaller offices, allowing remote working from home for several employees, and considering the possibility that employees come to the office on an alternating schedule. One of the main measures adopted by the Governments, to contain pandemic emergency, is to reduce the number of people sharing the same office/room. Indeed, to ensure the so-called social distancing which is a safe distance, the actual office/room capacity has been significantly reduced.

In this context, employees can be classified as essential employees and non-essential employees. Essential employees have a position designated as critical, because they have responsibilities of non-deferrable services, that must be performed despite an emergency; non-essential employees have positions non-vital during firms/universities closure. This last class of employees can work remotely from home, but they need appropriate devices and high-speed internet access. As underlined in [19], staff scheduling is a complex and time-consuming problem to deal with in any organization. In a highly changing situation, such as the current Covid-19 pandemic, several companies are forced to tackle the challenges associated with the adoption of flexible staff schedules. Indeed, it is not possible to build and use fixed workforce schedules over time, since employees’ availability varies due to either familiar responsibilities or because employees who test positive for Covid-19 cannot return to work until they have fully recovered.

The flexible work scheduling system, proposed in this paper, takes into account several employee’s features, such as employee’s contract, which also fixes the weekly work-hours, age, health status, and family conditions (i.e., parents of children should work at home because schools are closed, or some employees became family caregivers). It is assumed that employees express their availabilities and preferences for specific shifts of each work day and day off. In normal circumstances, the day off may be negotiated by employees and, usually, it is fixed for the entire year. During a pandemic, due to the highly dynamic employees’ availability, a flexible day off schedule is preferable to ensure continuity of the service. It is also important to note that the recommended anti-infection safety distance from other people led to re-evaluate the presence of employees in the same office/room and laboratories. To this aim, the office/room capacity is evaluated by considering anti-infection safety distance. Without loss of generality, we use a week as a planning horizon, in order both to deal with employees’ requests on an on-going fashion and to limit changes to the schedule.

We assume that each work day of a week is divided into $|{\mathcal {S}}|$ shifts and each shift $s \in {\mathcal {S}}$ has $h_s$ working hours. For now, we assume that the shifts are not overlapping and there is no incompatibility among them. The optimization model assigns work shifts with deference to features specified by the company, and with deference to employees’ availabilities/preferences. Several constraints concerning demand requirements, task skills specifications, contractual obligations, and employees’ preferences related to work on-site and/or to work at home are considered. A detailed description of the conditions that have to be taken into account is given in what follows.

$$H_1$$
The number of employees on duty in each shift-day is at least equal to the demand for employees on the shift-day.
$$H_2$$
Each employee cannot work in a day more than the maximum daily work hours, as specified by his/her contract of employment.
$$H_3$$
Each employee must work a given number of hours per week, as specified by his/her contract of employment.
$$H_4$$
Each employee fills in a schedule in order to specify unavailability at work in some shifts per each work day.
$$H_5$$
Employees take turns on-site.
$$H_6$$
The number of employees in an office during a shift-day cannot be greater than the office capacity.

## Mathematical models

This section is devoted to a detailed description of the proposed optimization models. We firstly show (see Sect. 3.1) the multiobjective nature of the problem under study and that an empty feasible region can exist, because of the null intersection of the hard constraints $H_1$–$H_6$, reported above.

Then, in Sect. 3.2 we develop a mixed integer linear programming (MIP) model with single objective and soft constraints. The main aim is to assign employees to shift-day in such a way that all the activities are carried out in an appropriate manner and the employees’ preferences are maximised. Three enhanced versions of this basic formulation are described in Sect. 3.3, whereas in Sect. 3.4, we present a more flexible variant, defined in such a way to handle the main characteristics of a Department of an Italian University, used as a case study.

In particular, we adopt a novel and flexible shift-day setting that allows employees to work, both on-site and remotely, respecting their family commitments, which vary over time. Indeed, the new setting presents more shifts in a day than the classical two daily shifts, i.e., Morning and Afternoon.

### A multi-objective optimization model

The first optimization model is a multi-objective problem (MOP), where all the constraints are of the hard type.

Let ${{\mathcal {D}}}$ be the set of days over the planning period; ${{\mathcal {S}}}$ be the set of shifts (e.g., morning, afternoon); ${\mathcal {K}}$ be the set of jobs, and ${\mathcal {E}}$ be the set of employees. Let $x_{eds}$ be the binary decision variable that takes a value equal to 1 if the employee $e\in {{\mathcal {E}}}$ works on-site on day $d \in {{\mathcal {D}}}$, during the shift $s \in {{\mathcal {S}}}$, and $x_{eds}=0$ if the employee $e\in {{\mathcal {E}}}$ either does not work at all on day $d \in {{\mathcal {D}}}$ or works remotely. A similar meaning has the binary decision variable $y_{eds}$ referred to remote work.

**Table 1:** Sets, parameters, and decision variables

*Sets*	
${{\mathcal {D}}}$	Days over the planning period
${{\mathcal {S}}}$	Set of shifts (e.g., morning, afternoon)
${\mathcal {R}}$	Set of room-office and laboratories
${\mathcal {K}}$	Set of jobs
${\mathcal {E}}$	Set of employees
${{\mathcal {E}}}{^0} \subseteq {\mathcal {E}}$	Set of employees that worked the past planned periods on-site. These employees should not work on-site in the planning period.
*Parameters*	
$C_{dsk}\ge 1$	Minimum staff required on-site for job *k* on shift *s* of day *d*
$t_{ds}$	Time slot duration of slot *s* on day *d*
$cap_r \ge 1$	Room/office capacity
$a_k=1$	If job *k* has alternating employees on-site; 0, otherwise
*W*	Matrix $|{{\mathcal {E}}}|$x$|{{\mathcal {D}}}|$x$|{{\mathcal {S}}}|$. It is used to take in consideration when an employee is available over the planning horizon
*RW*	Matrix $|{{\mathcal {E}}}|$x$|{{\mathcal {D}}}|$x$|{{\mathcal {S}}}|$. It is used to take in consideration when an employee is available to work remotely over the planning period
*For each employee in* ${{\mathcal {E}}}$	
$h_e$	Overall number of work hours over the planning period
$mdh_e$	Maximum daily work hours
$sk_e \in {\mathcal {K}}$	Employee’s job
$ar_e \in {{\mathcal {R}}}$	Assigned room/office
${\bar{d}}_e \in {{\mathcal {D}}}$	preferred day off
$wr_e=$	0, if employee *e* must work only on-site;
	1, if employee *e* must work only remotely;
	2, if employee *e* can work either on-site or remotely
*Decision variables*	
$x_{eds}=$	1, if employee $e\in {{\mathcal {E}}}$ works on-site on day $d \in {{\mathcal {D}}}$, shift $s \in {{\mathcal {S}}}$, 0 otherwise
$y_{eds}=$	1, if employee $e\in {{\mathcal {E}}}$ works remotely on day $d \in {{\mathcal {D}}}$, shift $s \in {{\mathcal {S}}}$, 0 otherwise

To formulate the MOP model, we require the notation provided in Table 1. The sets ${\mathcal {D}}, E, S, K$ are indexed by *d*, *e*, *s*, *k*, respectively. To simplify the model, let ${{\mathcal {E}}}_k{^0}$ denote the set of employees, performing job *k*, that worked the last planned week. In particular, these employees worked on-site in the past planned periods and should not work in the current planning period, because employees should take turns. The set ${{\mathcal {E}}}_k{^0}$ is updated offline before to plan the next period and it is used to assure alternating schedules for employees that work in the office. The matrices *W* and *RW* are utilized to represent the employee availability over the planning horizon. Indeed, the generic element of *W* is denoted by $w_{eds}$: $w_{eds}=1$ if employee *e* is available to work on shift *s* of day *d*; $w_{eds}=0$ otherwise. An element of matrix *RW* is denoted by $rw_{eds}$: $rw_{eds}=1$, if employee *e* wants to work remotely on shift *s* of day *d*; $rw_{eds}=0$, if employee *e* wants to work on-site on shift *s* of day *d*. A shift $s \in {{\mathcal {S}}}$ has a defined starting time and a defined length. For now, we assume that the daily shifts do not overlap. However, as we show in Sect. 3.4, different shifts, even if they may overlap, enable better schedules and improve employees satisfaction.

The defined formulation can be represented mathematically as reported in what follows:

1 $$\begin{aligned}&f_1= \max \sum \limits _{e\in {{\mathcal {E}}}} \sum \limits _{d\in {{\mathcal {D}}}} \sum \limits _{s\in {{\mathcal {S}}}} x_{eds} \end{aligned}$$

2 $$\begin{aligned}&f_2= \max \sum \limits _{e\in {{\mathcal {E}}}} \sum \limits _{d\in {{\mathcal {D}}}} \sum \limits _{s\in {{\mathcal {S}}}} y_{eds} \end{aligned}$$

3 $$\begin{aligned}&x_{eds}+ y_{eds} \le 1&\forall e \in {{\mathcal {E}}}, d \in {{\mathcal {D}}}, {s\in {{\mathcal {S}}}} \end{aligned}$$

4 $$\begin{aligned}&y_{eds} =0&\forall e \in {{\mathcal {E}}}|wr_e=0, d \in {{\mathcal {D}}}, {s\in {{\mathcal {S}}}} \end{aligned}$$

5 $$\begin{aligned}&x_{eds} =0&\forall e \in {{\mathcal {E}}}|wr_e=1, d \in {{\mathcal {D}}}, {s\in {{\mathcal {S}}}} \end{aligned}$$

6 $$\begin{aligned}&\sum \limits _{d\in {{\mathcal {D}}}} \sum \limits _{s\in {{\mathcal {S}}}} t_{ds}(x_{eds}+y_{eds}) = h_e&\forall e \in {{\mathcal {E}}} \end{aligned}$$

7 $$\begin{aligned}&\sum \limits _{s\in {{\mathcal {S}}}} t_{ds}(x_{eds}+y_{eds}) \le mdh_e&\forall e \in {{\mathcal {E}}}, d\in {{\mathcal {D}}} \end{aligned}$$

8 $$\begin{aligned}&\sum \limits _{e\in {{\mathcal {E}}}\mid sk_e=k} x_{eds} \ge C_{dsk}&\forall {d\in {{\mathcal {D}}}}, {s\in {{\mathcal {S}}}}, k \in {{\mathcal {K}}} \end{aligned}$$

9 $$\begin{aligned}&\sum \limits _{e\in {{\mathcal {E}}}\mid ar_e=r} x_{eds} \le cap_r&\forall {d\in {{\mathcal {D}}}}, {s\in {{\mathcal {S}}}}, r \in {{\mathcal {R}}} \end{aligned}$$

10 $$\begin{aligned}&x_{eds} \le w_{eds}&\forall { e\in {{\mathcal {E}}}, d\in {{\mathcal {D}}}, s\in {{\mathcal {S}}}} \end{aligned}$$

11 $$\begin{aligned}&y_{eds} \le w_{eds}&\forall {{e\in {{\mathcal {E}}}, d\in {{\mathcal {D}}}}, {s\in {{\mathcal {S}}}}} \end{aligned}$$

12 $$\begin{aligned}&y_{eds} \le rw_{eds}&\forall {{e\in {{\mathcal {E}}}, d\in {{\mathcal {D}}}}, {s\in {{\mathcal {S}}}}} \end{aligned}$$

13 $$\begin{aligned}&x_{eds} =0&\forall {{e\in {{\mathcal {E}}}^0 \mid wr_e=2, d\in {{\mathcal {D}}}}, {s\in {{\mathcal {S}}}}} \end{aligned}$$

14 $$\begin{aligned}&x_{eds}, y_{eds} \in \{0,1\}&\forall e \in {{\mathcal {E}}},d \in {{{\mathcal {D}}}}, s\in {{\mathcal {S}}} \end{aligned}$$

The proposed MOP is characterized by two objective functions: $f_1$ aimed at maximizing employee work on-site; $f_2$ aimed at maximizing remote work. Note that the objective function (1) is introduced to ensure a high level of efficiency, even during the Covid-19 pandemic. Constraints (3) state that each employee can work either in presence or remotely in every shift-day, or that he/she is not working. Constraints (4) impose that some employees cannot work remotely, as well as, Constraints (5) impose that some employees cannot work on-site. Constraints (6) state that the overall work hours over the planning horizon are equal to the amount specified in the employee contract, whereas Constraints (7) assure that the daily work hours are not greater than the maximum allowed. Constraints (8) are coverage constraints that make sure that each shift request is fulfilled. Constraints (9) state that the number of employees in a room/office cannot exceed the office capacity, which is determined by considering anti-infection safety distance. Constraints (10)–(12) avoid shift-day assignments that an employee discarded. Constraints (13) assure turnover for the nest planning period. Finally, Constraints (14) define the binary decision variables.

It is worth observing that the variables $x_{eds}$ and $y_{eds}$ are not complementary, that is, an employee may not work at all on a shift of a working day. The multi-objective model tries to find a schedule that matches employees’ availability and work requests. The two objectives are conflicting because when the number of employees working on-site, to be maximized, increases (improves), the number of employees working remotely decreases (deteriorates).

Another observation about Model (1)–(14) is that the hard constraints, mainly Constraints (10)–(12), could make the problem infeasible. Thus, this multi-objective model points out that a rigid organization could be not feasible in a pandemic situation. In order to overcome this critical issue, in Sect. 3.2 we present another mathematical formulation, in which soft constraints are considered and penalty terms are added directly to the objective function. In addition, we propose a more flexible organization, described in Sects. 3.3 and 3.4, and develop single-objective optimization models of increasing flexibility and complexity.

### A MIP model with soft constraints

Instead of solving the MOP (1)–(14), we formulate a MIP model, where only one objective function is optimized and soft constraints, with associated penalty weights, are introduced. The soft constraints can be violated. Nevertheless, their violations are penalised in the objective function. Instead of employee’s availability at work, we consider employee’s preferences. Requests of employees with specific needs (i.e., age, health or family conditions) are handled first. Employee’s needs, such as unavailability to work in some time periods, preferences for specific shifts in some days, are respected as possible. The hard constraints $H_5$ are removed and satisfactions of employee’s needs and alternation at office of employees are formulated as the following soft constraints:

$$S_1$$
Not satisfied employee’s needs (i.e., unavailability to work in some time periods, preferences for a specific shift type in some days) are penalised in the objective function.
$$S_2$$
Employees take turns on-site. If this condition is not satisfied, there is a penalty cost in the objective function.

To formulate the first MIP model with soft constraints (referred in the sequel as $Model_1$) new parameters and decision variables are introduced, as reported in Tables 2 and 3, respectively.

**Table 2:** Added parameters of the MIP models

Parameters	
*W*	Matrix $|{{\mathcal {E}}}|$x$|{{\mathcal {D}}}|$x$|{{\mathcal {S}}}|$. It is used to take in consideration employee’s preferred day-shift assignments over the planning period
*RW*	Matrix $|{{\mathcal {E}}}|$x$|{{\mathcal {D}}}|$x$|{{\mathcal {S}}}|$. It is used to take in consideration employee’s preferred day-shift assignments to work remotely over the planning period
${\bar{M}}_1>0$	A weight to penalize the violation of employee’s preference regarding the shift assignments
${\bar{M}}_2>0$	A weight to penalize the violation of employee’s preference regarding the shift assignments to work remotely
${\bar{M}}_3>0$	A weight to penalize the violation of employee’s preferred day off
$c \in \{0,1\}$	A cost coefficient used to select one of the two first terms of the objective function

**Table 3:** Added variables

Decision and auxiliary variables	
$z_{eds}=1$,	If employee’s preference, who would prefer not to work on day *d* and shift $s \in {{\mathcal {S}}}$, is not respected, 0 otherwise
$z^{'}_{eds}=1$,	If employee’s preference, who would prefer remote work on day *d* and shift $s \in {{\mathcal {S}}}$, is not respected, 0 otherwise
$ne_{d}\ge 0$,	Number of employees working on day *d* on-site
${\tilde{x}}_{ed}=1$,	If employee $e \in {\mathcal {E}}$ works on-site at least during one shift of day *d* , 0 otherwise
${\bar{x}}_e=1$,	If employee $e \in {\mathcal {E}}$ works on-site, 0 otherwise

On the basis of the notation introduced above, $Model_1$ can be represented mathematically as follows:

15 $$\begin{aligned} \max \ \&\sum \limits _{d\in {{\mathcal {D}}}} ne_d - {{\bar{M}}_1} \sum \limits _{e\in {{\mathcal {E}}}} \sum \limits _{d\in {{\mathcal {D}}}} \sum \limits _{s\in {{\mathcal {S}}}} z_{eds} - {{\bar{M}}_2} \sum \limits _{e\in {{\mathcal {E}}}} \sum \limits _{d\in {{\mathcal {D}}}} \sum \limits _{s\in {{\mathcal {S}}}}z^{'}_{eds}-{{\bar{M}}_3} \sum \limits _{e\in {{\mathcal {E}}}} \sum \limits _{s\in {{\mathcal {S}}}} z_{e{\bar{d_e}}s} \end{aligned}$$

Constraints (3)–(9)

16 $$\begin{aligned}&{\tilde{x}}_{ed} \ge x_{eds}&\forall e\in {{\mathcal {E}}}, {d\in {{\mathcal {D}}}}, {s\in {{\mathcal {S}}}} \end{aligned}$$

17 $$\begin{aligned}&{\tilde{x}}_{ed} \le \sum \limits _{s\in {{\mathcal {S}}}}x_{eds}&\forall e\in {{\mathcal {E}}}, {d\in {{\mathcal {D}}}}\end{aligned}$$

18 $$\begin{aligned}&ne_{d} = \sum \limits _{e\in {{\mathcal {E}}}} {\tilde{x}}_{ed}&\forall {d\in {{\mathcal {D}}}} \end{aligned}$$

19 $$\begin{aligned}&z_{eds}\ge x_{eds}&\forall {d\in {{\mathcal {D}}}}, {s\in {{\mathcal {S}}}}, k \in {{\mathcal {K}}}|a_k=1, \nonumber \\&e\in {{\mathcal {E}}}\mid e \in {{\mathcal {E}}}_k^{0} \end{aligned}$$

20 $$\begin{aligned}&z_{eds}\ge (x_{eds}+y_{eds})(1-w_{eds})&\forall e \in {{\mathcal {E}}}, d \in {{\mathcal {D}}}, {s\in {{\mathcal {S}}}} \end{aligned}$$

21 $$\begin{aligned}&z^{'}_{eds}\ge x_{eds} rw_{eds}&\forall e \in {{\mathcal {E}}} \mid wr_e=3, d \in {{\mathcal {D}}}, {s\in {{\mathcal {S}}}} \end{aligned}$$

22 $$\begin{aligned}&z^{'}_{eds}\ge y_{eds} (1-rw_{eds})&\forall e \in {{\mathcal {E}}} \mid wr_e \ge 2, d \in {{\mathcal {D}}}, {s\in {{\mathcal {S}}}} \end{aligned}$$

23 $$\begin{aligned}&x_{eds},y_{eds},z_{eds}, z^{'}_{eds} \in \{0,1\}&\forall e \in {{\mathcal {E}}},d \in {{{\mathcal {D}}}}, s\in {{\mathcal {S}}} \end{aligned}$$

24 $$\begin{aligned}&{\tilde{x}}_{ed} \in \{0,1\}&\forall e \in {{\mathcal {E}}},d \in {{{\mathcal {D}}}} \end{aligned}$$

The objective function (15) is an algebraic sum of four terms. The first term maximizes the overall number of employees that work on-site during the planning period. The second and the third terms take into account unsatisfied employee’s preferences, which are computed by Constraints (19)–(22). The last term penalises unsatisfied preferred day off works. Observe that every shift assigned during the preferred day off is penalised.

Constraints (16)–(17) set the value of the binary variables ${\tilde{x}}_{ed}$, which are used to know if an employee works on day *d*. Constraints (18) define $ne_d$ as the overall number of different employees working on-site on day *d*. Constraints (19) are related to those employees that take turns and that worked in the past periods: if an employee of this set, who should not work on-site over the planning period is again scheduled to some shift to work in the office, the related auxiliary variables are set to one. These violations are even penalised in the second term of the objective function.

Constraints (20) are on unsatisfied employee’s working shift-day preferences, whereas Constraints (21)–(22) are on unsatisfied remote working shift-day employee’s preferences. As an example, let ${{\mathcal {S}}}=\{M, A\}$ be the set of shifts, i.e., morning shift and afternoon shift. Assume that an employee would like to work on Monday afternoon only remotely. If he/she must work remotely on Monday morning and in office on Monday afternoon, then $z^{'}_{e1A}=1$ because of Constraints (21) and $z^{'}_{e1M}=1$ because of Constraints (22). The third term of the objective function penalises these two kinds of non-preferred shift-day assignments. Finally, Constraints (23)–(24) are on the decision variables.

$Model_1$ (15)–(24) searches for feasible schedules as trade-off among competing goals. Note that finding an optimal solution means that the determined schedule is acceptable for a decision-maker. In order to guarantee constraint satisfaction, the penalty values have to be chosen large enough such that slack variables are kept at zero, if possible.

### Enhanced optimization models

In this section, three enhanced versions of $Model_1$ are described. Their main aim is to ensure the fulfillment of the coverage requirements and employee satisfaction at the same time. The first formulation, referred to as $Model_{2a}$, is aimed at limiting the number of different employees that daily work on-site; the second model, referred to as $Model_{2b}$, is aimed at limiting the number of different employees that work on-site during the planning period. Finally, $Model_{2c}$ handles specific situations in which the number of different employees that work on-site during the planning period should be either maximised or minimised.

$Model_{2a}$ aims to fulfill coverage requirements and employee’s needs and to reduce the number of different employees that work on-site every day, at the same time. In this model, the term $\sum \limits _{d\in {{\mathcal {D}}}} ne_d$ of the objective function (15) is replaced by:

$$\begin{aligned} \min \max _{d\in {{\mathcal {D}}}} \sum \limits _{e\in {{\mathcal {E}}}} \sum \limits _{s\in {{\mathcal {S}}}} x_{eds}. \end{aligned}$$

This function is non linear, but can be linearized by introducing the following constraints:

25 $$\begin{aligned} ne_{d} \ge \sum \limits _{e\in {{\mathcal {E}}}} \sum \limits _{s\in {{\mathcal {S}}}} x_{eds} \quad \forall d\in {{\mathcal {D}}}. \end{aligned}$$

The objective function (15) is then replaced by:

26 $$\begin{aligned} \min \ \&ne_{d} + {{\bar{M}}_1} \sum \limits _{e\in {{\mathcal {E}}}} \sum \limits _{d\in {{\mathcal {D}}}} \sum \limits _{s\in {{\mathcal {S}}}} z_{eds} + {{\bar{M}}_2} \sum \limits _{e\in {{\mathcal {E}}}} \sum \limits _{d\in {{\mathcal {D}}}} \sum \limits _{s\in {{\mathcal {S}}}}z^{'}_{eds}+{{\bar{M}}_3} \sum \limits _{e\in {{\mathcal {E}}}} \sum \limits _{s\in {{\mathcal {S}}}} z_{e{\bar{d_e}}s}&\end{aligned}$$

and Constraints (16)–(18), and Constraints (24) are removed.

To formulate $Model_{2b}$, we introduce new decision variables and new constraints, as follows. Let ${\bar{x}}_{e}$, be a binary variable that takes value one if employee *e* works on-site at least one time during the planning period, zero otherwise. We add the following constraints:

27 $$\begin{aligned}&{\bar{x}}_{e} \ge x_{eds}&\forall e \in {{\mathcal {E}}}, d \in {{\mathcal {D}}}, s \in {{\mathcal {S}}} \end{aligned}$$

28 $$\begin{aligned}&{\bar{x}}_{e} \le \sum \limits _{d\in {{\mathcal {D}}}} \sum \limits _{s \in {{\mathcal {S}}}} x_{eds}&\forall e \in {{\mathcal {E}}} \end{aligned}$$

29 $$\begin{aligned}&\sum \limits _{e\in {{\mathcal {E}}}\mid ar_e=r} {\bar{x}}_{e} \le cap_r&\forall r \in {{\mathcal {R}}} \end{aligned}$$

30 $$\begin{aligned}&{\tilde{x}}_{e} \in \{0,1\}&\forall e \in {{\mathcal {E}}} \end{aligned}$$

Constraints (27)–(28) define the value for the binary variables ${\bar{x}}_{e}$. More specifically, if an employee *e* is assigned at least to one shift during the planning period, i.e., *e* has to work on-site at least one time, then $\sum \nolimits _{d\in {{\mathcal {D}}}} \sum \nolimits _{s \in {{\mathcal {S}}}} x_{eds} \ge 1$. As a consequence, the binary variable ${\bar{x}}_{e}=1$ because of Constraints (27)–(28). Constraints (29) limit the number of employees present in a room/office at any given time, over the whole planning horizon, ensuring that it is no more than $cap_r$. For instance, if $cap_r=2$ for a given room *r*, the number of different employees that could work on-site in room/office *r* during the planning horizon is only two. Constraints (30) define the binary variables.

$Model_{2b}$ is obtained from $Model_{2a}$ by adding Constraints (27)–(28) and Constraints (30); by removing Constraints (25); by replacing Constraints (9) with Constraints (29), and the first term of the objective function (26) with

31 $$\begin{aligned} \ \&\sum \limits _{{e\in {{\mathcal {E}}}}} {\bar{x}}_e. \end{aligned}$$

Thus the objective function of $Model_{2b}$ assumes the following form:

32 $$\begin{aligned} \min \ \&\sum \limits _{{e\in {{\mathcal {E}}}}} {\bar{x}}_e + {{\bar{M}}_1} \sum \limits _{e\in {{\mathcal {E}}}} \sum \limits _{d\in {{\mathcal {D}}}} \sum \limits _{s\in {{\mathcal {S}}}} z_{eds} + {{\bar{M}}_2} \sum \limits _{e\in {{\mathcal {E}}}} \sum \limits _{d\in {{\mathcal {D}}}} \sum \limits _{s\in {{\mathcal {S}}}}z^{'}_{eds}+{{\bar{M}}_3} \sum \limits _{e\in {{\mathcal {E}}}} \sum \limits _{s\in {{\mathcal {S}}}} z_{e{\bar{d_e}}s}.&\end{aligned}$$

A further enhanced variant of $Model_{2b}$ can be formulated to represent opposite scenarios, in which the number of employees working on-site can be either maximized by adopting physical distancing measures or minimized in order to strongly reduce the risk of exposure to Covid-19. This more flexible model, referred as $Model_{2c}$, can be mathematically handled by introducing the following objective function:

33 $$\begin{aligned} \max \ \&c \sum \limits _{{e\in {{\mathcal {E}}}}} {\bar{x}}_e + (1-c) ( \vert {{\mathcal {E}}}\vert -\sum \limits _{{e\in {{\mathcal {E}}}}} {\bar{x}}_e)&\nonumber \\&-{{\bar{M}}_1} \sum \limits _{e\in {{\mathcal {E}}}} \sum \limits _{d\in {{\mathcal {D}}}} \sum \limits _{s\in {{\mathcal {S}}}} z_{eds} - {{\bar{M}}_2} \sum \limits _{e\in {{\mathcal {E}}}} \sum \limits _{d\in {{\mathcal {D}}}} \sum \limits _{s\in {{\mathcal {S}}}} z^{'}_{eds}- {{\bar{M}}_3} \sum \limits _{e\in {{\mathcal {E}}}} \sum \limits _{s\in {{\mathcal {S}}}} z^{'}_{e{{\bar{d}}}_es}&\end{aligned}$$

where *c* is a scalar that can assume only two values, that is 1 and 0. In particular, *c* is set equal to 1 if the goal is to maximize the number of on-site employees, whereas *c* is set equal to 0 if the objective function seeks the maximization of the overall number of employees that work remotely.

For the sake of clarity, Table 4 reports, for each proposed optimization model described in this subsection, the objective function, and the constraints.

**Table 4:** Objective function and constraints of the single-objective optimization models

Model	Obj. function	Constraints
$Model_1$	(15)	(3)–(9); (16)–(24)
$Model_{2a}$	(26)	(3)–(9); (19)–(23); (25);
$Model_{2b}$	(32)	(3)–(8); (19)–(23); (27)–(30)
$Model_{2c}$	(33)	(3)–(8); (19)–(23); (27)–(30)

### A flexible work-day schedule

Aware that home is not the office, we have proposed to the chair of the university’s department used as case study to modify the current work-day structure, which is traditionally partitioned into one Morning shift, which starts at 8.00, and one Afternoon shift, which starts at 15.00. We introduce new working shifts that can vary in terms both of starting-time and length. The aim of this re-organization is to help employees to make their tasks effectively and support them during this pandemic era, avoiding discriminatory and no-suitable schedules. In order to facilitate employees with children, for instance, and in general employees with family responsibilities, we propose to introduce shifts with a different start hour and a duration of 2 or 3 work hours. More specifically, there are two shifts in the morning and three in the afternoon. The proposed structure of a work-day in more than two shifts is such that at least one feasible solution exists. That is, there is at least one combination of shifts in each work-day such that the sum of their lengths is equal to $h_e$, for every employee *e*.

**Fig. 1:**
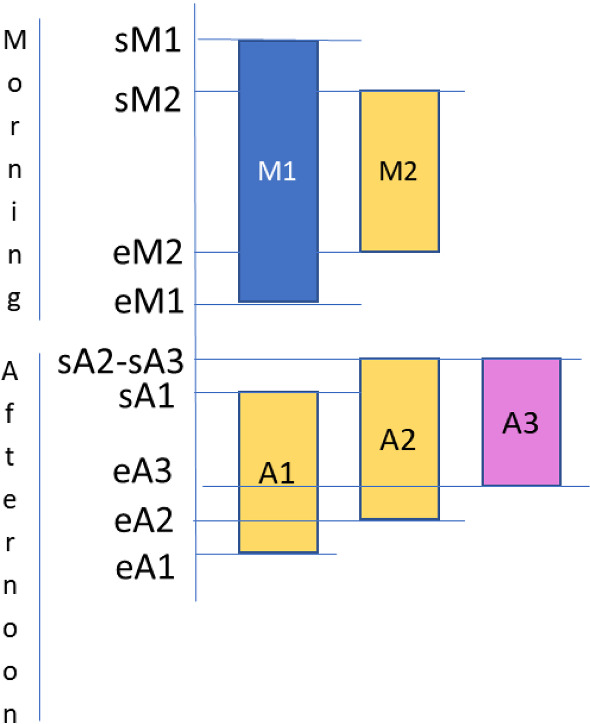
Graphical representation of a day with five work shifts. $sM_i$ and $sA_j$ denote the start time of i-th and j-th time slot in the morning and afternoon, respectively; $eM_i$ and $eA_j$ denote their end time, respectively

Figure 1 shows a graphical representation of the proposed shifts: $M_1$ and $M_2$ are the two shifts on the morning, and $A_1, A_2$ and $A_3$ the three shifts defined in the afternoon. These shifts differ for start time and duration, as detailed in Table 5.

**Table 5:** Start time and end time of the daily work shifts

	$M_1$	$M_2$	$A_1$	$A_2$	$A_3$
Start time	8:00	9:30	15:00	14:30	14:30
End time	14:00	12:30	18:00	17:30	16:30

Overlapping shifts and specific shift combinations cannot be assigned. For instance, $M_1$ and $M_2$ are overlapping shifts on a day and they cannot be assigned to an employee, as well as $M_1$ with $A_2, A_3$ cannot be assigned because an employee must have 1-hour lunch break, and $A_2, A_3$ because they are overlapping shifts. In order to simplify the notation, let ${{\mathcal {S}}}=\{1,\ldots , 5\}$, be the set of shifts that are ordered as reported in Fig. 1, from left to right and from up to bottom. Let *O* be a set of shift-assignments mutually incompatible, and ${\mathcal {O}}$ be the set of incompatible sets *O*. For the real case study, let $O_1=\{1,2\}, O_2=\{1,4,5\}, O_3=\{3,4,5\}$, be the three sets of incompatible shift-assignments. A feasible assignment for each $i, k \in {{\mathcal {S}}}$ should satisfy the following condition $i \in O_j, k \in O_l, k \notin O_j, j,l=1,\ldots , 3.$ This means that, for instance, (1, 4) as well as (3, 5) are forbidden shift-assignments. There are thus six forbidden shift-assignments.

In order to handle the peculiarities of the case study, the following sets of constraints are added to $Model_{2c}$:

34 $$\begin{aligned}&\sum \limits _{s\in {O_j}} x_{eds}+ y_{eds} \le 1&\forall j=1\ldots 3, e \in {{\mathcal {E}}}, d \in {{\mathcal {D}}} \end{aligned}$$

35 $$\begin{aligned}&x_{ed1} \ge x_{ed3}&\forall e \in {{\mathcal {E}}}, d \in {{\mathcal {D}}} \end{aligned}$$

Constraints (34) avoid inconsistent shift assignments. Constraints (35) model the department’s request, that is, an employee that works in $A_1$ must also work in $M_1$. In what follows, this formulation is referred to as $Model_{3}$.

## Computational experiments

In this section, we present the computational results obtained by solving the most flexible versions of the proposed mathematical models (i.e., $Model_{2c}$ and $Model_{3}$). The experimentation is divided in two main phases. In the first one, $Model_{2c}$ is solved on real data provided by the Department of Mechanical, Energy and Management Engineering (DIMEG, *www*.*dimeg*.*unical*.*it*), University of Calabria, Italy. The main aim is to evaluate the impact of a high level of flexibility on the solution quality. In the second phase, the computational experiments are carried out on $Model_{3}$ by considering even larger and realistic instances with different characteristics. The main purpose is to evaluate the performance of the model in terms of scalability. The models have been coded in OPL and solved with CPLEX 12.7.1, on an Intel Core i7-3632QM, 2.20GHz with 8GB RAM, running under the operating system Windows 10 Pro. The data sets generated and analysed during the current study are available from the corresponding author on reasonable request.

### Numerical results on the real case study

We have considered as case study the technical and administrative staff scheduling at the DIMEG,University of Calabria, Italy.

The University of Calabria (UniCal, *www*.*unical*.*it*), named for the region in the south of Italy, is a residential campus university, organized in fourteen departments. In the Covid-19 emergency, UniCal has adopted a work-from-home policy and the virtual private network (VPN) is used to connect remote workers to the university’s internal network. The manual scheduler of a department works in a highly dynamic mode, because there can be several requests for changes. For instance, frequently employee’s availability changes, mainly for those employees with children. Producing a schedule manually, taking into account dynamic employee’s availability and preferences, and thus matching all requirements, is very difficult and time consuming. The DIMEG is planning suitable schedules even for employees taking turns at university and employees working from home. The department staff duties concern administrative work, information technology, mechanical laboratories. We collected real data of DIMEG’s employees, summarised as follows.

*Department requirements* The adopted planning period is a week, from Monday to Friday. Each day has currently two shifts given as 8:00 a.m. – 2:00 p.m. and 3:00 p.m. – 6:00 p.m. The daily required number of employees working at university per each job is at least one in the morning. To reduce sanitizing costs and limit the number of different employees working over a week, the department has decided that the same employees work for the entire week in his/her office. In case of employees that take turns, an office is sanitised during the weekend.

*Office capacity* The employees are located in room/office and laboratories. Four offices have a reduced capacity to one for emergency Covid-19, and this implies employees that take turns if they work at university. Since the fragile employees must work remotely, new office assignments have been carried out in order to ensure employees safety if they work at university.

**Fig. 2:**
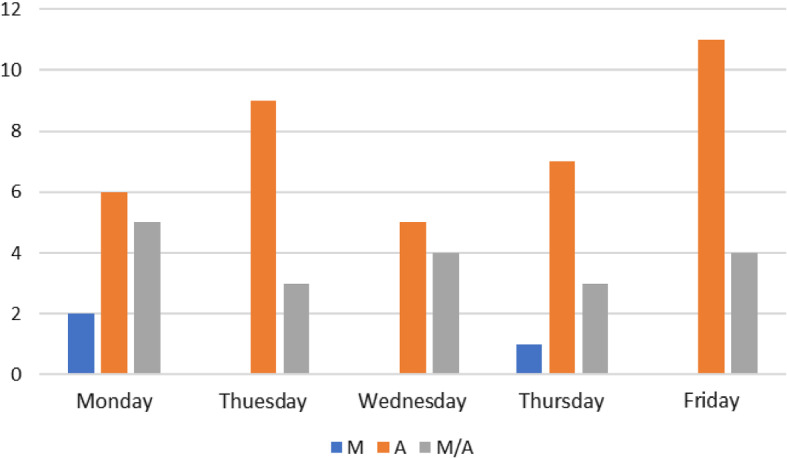
Distribution of employees’ unavailability to work on Morning (M), on Afternoon (A), and both Morning and Afternoon (A/M)

*Employees’ needs and preferences* The overall number of employees is twenty-two, all are full-time, and they perform different jobs. Their labor contract includes 36 hours per week and no more than 9 hours per day. Three employees have a non-deferrable position. In order to obtain information about the individual availability, the personal needs and preferences, in reference to morning and afternoon shift per day and preferred day off, a questionnaire was administered to all staff members. The main findings of the survey were that five employees have children and should take care of them and three employees are classified as fragile (for age or some pathologies). Figure 2 depicts how employees unavailability to work on the Morning shift and Afternoon shift per day are distributed. Observe that the majority of them should not work on Friday afternoon.

As better described below, a second questionnaire was administered to all staff members in order to know their preferences regarding the proposed flexible shift-day organisation.

We solved $Model_{2c}$ twice, i.e., with $c=0$ and $c=1$, obtaining two different schedules for the DIMEG staff. A schedule is given as a sequence of working days and days-off for each employee and it covers all the time horizon.

The solution has been obtained in less than two seconds.

We set the three parameters that penalise employee’s availability and preference as ${\bar{M}}_1=10, \ {\bar{M}}_2=5, \ {\bar{M}}_3=100$, respectively. ${\bar{M}}_1>{\bar{M}}_2$ because ${\bar{M}}_1$ refers to assignments on shift-day in which employee would not want to work; ${\bar{M}}_3>{\bar{M}}_1$ because it strongly penalises assignments on shift-day of the chosen day-off. Employee’s preferences are related to Morning and Afternoon shift. The minimum number of employees with a job can be different in each shift-day and week by week. However, following the department requirements for Morning shifts, we set $C_{d1k}=C_{d2k}=1, \forall d\in {{\mathcal {D}}}, \forall k\in {{\mathcal {K}}}.$ Employees $E_1,E_{15},E_{16}$ have to work in the mechanical laboratories and for them the parameter $wr_e$ is set equal to 0 (as reported in Table 1); employees $E_2,E_3,E_4, E_{9},E_{14},E_{19},E_{20},E_{21}$ have to work only remotely (because of age, family responsibility, or health fragility) and the corresponding parameter $wr_e$ is set equal to 1, as reported in Table 1.

Two different schedules have been found, by letting firstly $c=1$ and then $c=0$ in the objective function (33). If we set the parameter of the objective function as $c=1$, the number of employees working on-site, which is maximised, is equal to twelve; if we set $c=0$, the maximum number of employees that work on-site is eight. Both the two schedules minimise the number of employees for whom some preferences are not met. However, some employees have to work on-site even during their chosen day-off. This happens because department requirement (i.e., minimum staff demand) and the safety measures for those employees that must work remotely, force to violate some day-off preferences.

**Fig. 3:**
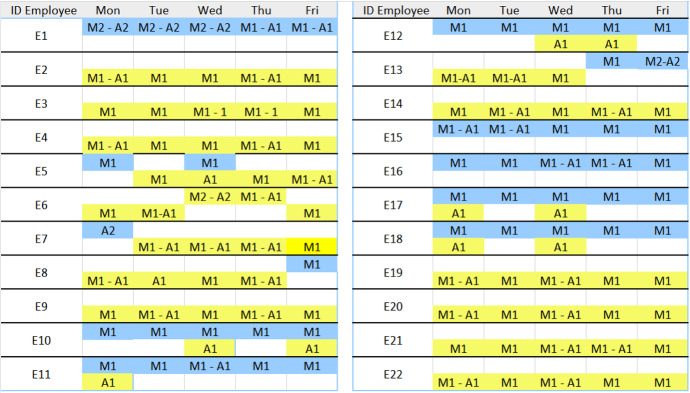
Schedule with $$c=1$$

**Fig. 4:**
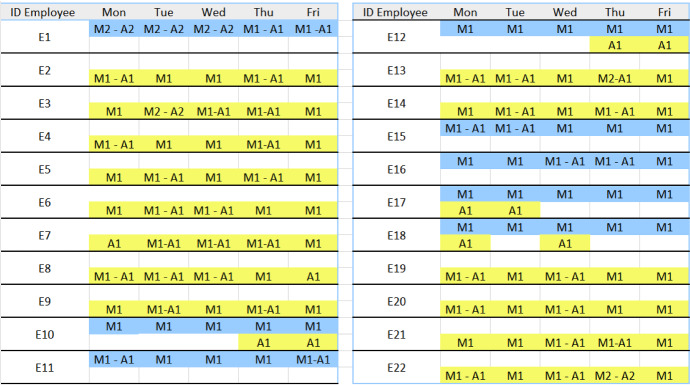
Schedule with $$c=0$$

Finally, we consider for the five employees that have children, work remotely and are not able to work traditional five-day, the possibility to implement a more flexible scheduling policy, where the assigned work shift $M_1$ is replaced by $M2-A_1$ or $M2-A_2$. The proposed flexible work-day organization allows to increase their satisfaction and support them in managing their work. The schedules are shown in Fig. 3 (for $c=1$) and Fig. 4 (for $c=0$). For each employee there are two rows: the first row is related to on-site work (black color), whereas the second row refers to remote work (yellow color). As the schedules in Figs. 3 and 4 show, for these employees the hard constraints are satisfied. These schedules, if accepted by the employees, can be used to update the set ${{\mathcal {E}}}^0$ that is the set of employees that should not work on-site the next planning period.

### Computational results on larger-size instances

Further computational experiments were carried out by solving $Model_3$ on eighth larger-size and realistic instances. This analysis is conducted with the aim of investigating the model’s scalability. The new instances differ for the overall number of employees, the number of employees that have to work on-site and those ones remotely, and the required minimum staff per job, as detailed in Table 6. Table 6 lists from the second to the fifth column the characteristics of the instances: the second column reports the overall number of employees, i.e., 22, 44, 120; the third column reports the number of employees that have to work only on-site. It is around 14% of the overall number of employees. The fourth column reports the number of employees that have to work only remotely; this number is set as 14% and 36% of the overall number of employees. The fifth column shows the required minimum staff per job, which is for simplicity assumed the same for all the jobs. There are 10 jobs. The number of rooms/offices and laboratories varies between a minimum of 18 and a maximum number equal to 43. We suppose that office capacity can be reduced to preserve employees healthy. The instances are based on two main scenarios

- Scenario 1: employees with children work only remotely as well as all fragile employees and those ones with non-deferrable family responsibilities, as family caregivers, for instance. This scenario is denoted by the letter *a* in the name of the computational experiment.
- Scenario 2: employees with children can work even on-site whereas fragile employees must work only remotely. This scenario is denoted by the letter *b* in the name of the computational experiment.

Scenario 1 occurs, for instance, when schools are closed. It is thus more restrictive with respect to the second one and strongly affects employees’ satisfaction.

In these experiments, we used the most flexible work-day organization that is based on the five shifts, as reported in Fig. 4. For that concerning employee’s preference for a shift-day, we asked the DIMEG staff to fill in a second questionnaire in order to know their preference. We use this real case instance as a basic instance and generated randomly employees’ preferences for the other instances. For each computational experiment, the sixth column shows the value of the parameter *c* of the objective function. The combination of scenario, instance characteristics, and value of the parameter *c* results in sixteen computational experiments, that can be grouped into four sets. The first column of Table 6 reports the name of a computational experiment. The seventh and eighth columns present the results in terms of absolute objective function value and the CPU time (in seconds), respectively.

**Table 6:** Computational results on realistic instances

Computational experiment	Number of employees	Empl. on-site employees	Remote employees	Minimum demand	c	Obj function	Time (seconds)
$Exp_{1a}$	22	3	8	1	1	1389	4.55
$Exp_{2a}$	22	3	8	1	0	1429	4.3
$Exp_{1b}$	22	3	3	1	1	927	4.48
$Exp_{2b}$	22	3	3	1	0	970	4.55
$Exp_{3a}$	44	6	16	2	1	2790	4.48
$Exp_{4a}$	44	6	16	2	0	2972	5.09
$Exp_{3b}$	44	6	6	2	1	2350	4.4
$Exp_{4b}$	44	6	6	2	0	2566	4.47
$Exp_{5a}$	44	6	16	2	1	2496	4.59
$Exp_{6a}$	44	6	16	2	0	2362	4.65
$Exp_{5b}$	44	6	6	2	1	1892	4.79
$Exp_{6b}$	44	6	6	2	0	2058	5.16
$Exp_{7a}$	120	17	43	3	1	3007	6.49
$Exp_{8a}$	120	17	43	3	0	3414	6.86
$Exp_{7b}$	120	17	16	3	1	3007	7.17
$Exp_{8b}$	120	17	16	3	0	3160	8.04

The first four rows refer to the DIMEG staff. All the found schedules minimise employees discomfort and the chosen day off is always respected with the only exception of $Exp_{1a}$ because the number of employees that work remotely impacts on the schedules of employees that have to work on-site. The computational time is very limited even when the largest instances, which have 120 employees, are solved. This time is not comparable to the excessive time needed to produce a schedule manually. Thus, the models can be used to support a staff manager efficiently.

A schedule can be implemented until changes in requirements or employees availability occur. Alternate work schedules can be generated by considering department’s needs and employees’ availability. An advantage of this approach is that it is possible instantaneously to update the schedule of the incoming week, modify a schedule if employee’s availability/preference changes and identify alternative schedules for employees if some shift-day assignments are not feasible for them.

## Conclusions

The scheduling of employees is a complex and time-consuming task, mainly if employees’ availability and preferences are considered. In this paper, we have presented integer linear programming models to address a currently emergent real-world problem, that is, staff scheduling in the pandemic Covid-19 era. We take into account demand requirements, employees’ personal and family responsibilities, and anti-Covid-19 measures. In order to improve staff scheduling and increase employee’ satisfaction and supporting employees, we propose a novel work-day organization based on more than the usual two shifts. The computational experiments were firstly carried out on real data provided by the Department of Mechanical, Energy and Management Engineering at University of Calabria. The found schedules are preferred with respect to those ones constructed by hand because they are obtained in few seconds, modified requests can be handle quickly and even a negotiation phase is less time consuming. In addition, the employee’s needs are better meet when the proposed work-day organization, based on several shifts with different duration and that may have overlaps, is used in comparison to the classical one. We carried out further tests on larger and randomly generated instances to measure the scalability. The results show that the model finds optimal schedules in short computing time even for larger instances.

The optimization models can be easily integrated in the university staff service and can be used in managing staff scheduling efficiently. It is worth observing that the models can be used not only for addressing the university staff scheduling problem, but also general staff scheduling problems in flexible settings.

Adopting a flexible work management offers numerous benefits to both employers and employees, as evidenced in our paper. First of all, it can be a solution for business continuity during emergency circumstances such as a pandemic. Other benefits include creating a better work/life balance for employees, enhancing their morale, managing employee attendance, reducing absenteeism, and boosting productivity.
